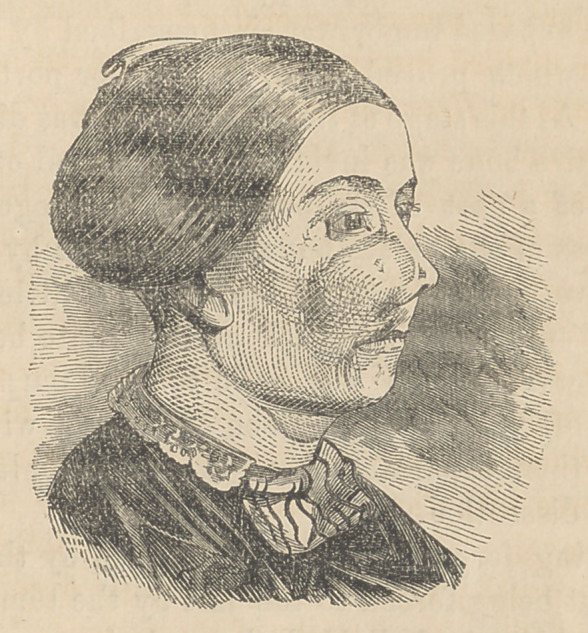# Pennsylvania College, Ninth below Locust Street

**Published:** 1852-12

**Authors:** W. H. Gobrecht


### CLINICAL REPORTS.

*Pennsylvania College, Ninth below Locust street. Service of* Professor GILBERT.

Reported by W. H. GOBRECHT, M. D.

*October* 23*d.*—CASE XXIII. *Resection of Superior Maxillary.*—In presenting a patient with Tumor of the Superior Maxillary bone, Prof. Gilbert made the following prefatory remarks upon the subject of tumors generally:

Tumors are divided into two great classes, viz.:—

1. *Malignant* or *Heterologous.* 2. *Benign* or *Analogous.*

The *Malignant*, in bones, are of two orders. 1. *Osteo-cephaloma*, which is a brain-like degeneration of the bone, whilst the surrounding tissues are healthy. 2. *Osteo-carcinoma*, where the affection is of a cancerous character.

The *Benign* has five species, viz.: 1. *Exostosis*, which is an outgrowth or extension of surface, and may be either *hard*—which occurs upon flat bones not often calling for removal—or *cancellated*, which, existing principally on the extremities of long bones, interferes with the action of the muscles and frequently demands removal. After these operations, inflammation and suppuration generally follow; we therefore dress with a dossil of lint, and heal from the bottom of the wound. 2. *Osteoma*, an enlargement of the entire circumference of the bone. This may degenerate, and then we remove, if possible, by amputation. 3. *Enchondroma.* This is a fibro-cartilaginous tumor connected with the osseous structures. Enchondromata are of two varieties. 1st. That developed within the substance or cavity of the bone, distending it. The 2d is dense and peripheral, growing from the periosteum and covered by a thin bony pellicle. 4. *Osteo-cystoma*, (of which spina ventosa is a form,) occurring in the lower jaw and in the long bones. In this form of disease we lay open the cyst and destroy it by cauterization. 5. *Osteo-sarcoma.* This is more difficult to distinguish from the malignant orders of tumors. It is a mixture of bony, cartilaginous and fleshy structures. A large and beautiful specimen of this description of disease, involving the upper part of the thigh and requiring amputation just below the hip joint, is preserved in the museum of this Institution.

The case before us is diagnosed to be an *Enchondroma.* The patient, Eliza McB——, of New Jersey, married, aged 32, states that about 11 years ago, a firm and hard swelling was noticed on the right side of the face, which slowly increased; latterly its growth has been more rapid, so that it now presents the enlarged appearance exhibited in the occompanying cut by Gihon, from a daguerreotype of Laughlin's.

The tumor, which is as hard as bone, involves the right superior maxillary, and extends from the base of the alveoli below to the orbital process above; is terminated externally by the malar process, and internally covers its nasal portion. The palatine plate is undisturbed, and the alveoli not involved; the nostril is not much obstructed, the tears passing freely into it, whilst the breathing is good. The eye is but slightly irritated. Very little pain exists. There is no dyscrasia, no lemon tint of the skin. The patient is neither wan nor spare. The age is favorable, and all these, together with the firmness, slowness of growth and small size, indicate most positively that its nature is *benign.* Hence we may safely state that there will be no return of the disease.

In all probability it is an *Enchondroma* springing from the anterior surface of the superior maxillary, but in going into the operation, we shall be prepared to remove the entire bone, should we discover it to be involved. Prof. Gilbert states that he has removed the superior maxillary entirely in one case, and the maxillary and malar bones in another, within the last eighteen months.

The patient being seated, an incision was made from near the inner canthus of the eye to the angle of the mouth, and the flaps thus formed dissected up from their attachments, the ala of the nose being separated from the side of the anterior naris. These flaps being then held aside by copper spatulæ and the fingers of assistants, the exposed tumor was circumscribed by a strong scalpel, and (that which proved to be) its anterior portion partially, broken away. At this stage of the dissection it was discovered that nearly the entire bone was involved, the removal of which (with the exception of a part of its posterior wall,) together with the palatine process of the palate bone, was effected by first dividing the tissues investing the roof of the mouth a little to the right of the mesial suture, as far back as the palate bone, at which point a transverse incision was made for the purpose of saving the attached soft palate. The globe of the eye, with its inferior muscles, was then separated from the orbital plate by careful elevation and dissection.

Strong cutting forceps were then applied by the mouth and right naris, but being forced to the left by the tumor, dislocated the septum narium and divided the palatine arch and alveoli on that side of the nasal crest. The malar process was now divided on a line extending into the anterior extremity of the spheno-maxillary fissure with Heys' saw and the bone nippers. The nasal process was next divided by pointed forceps high up, and the ethmoido-maxillary suture opened with a strong scalpel. The osseous connexions were now entirely divided, by drawing the superior maxillary forwards, and all adhering soft tissues detached, carefully preserving the continuity of the lateral half arch of the palate.

The hæmorrhage was not alarming, no ligatures being employed during the operation.

The actual cautery was now applied to every part of the cut surfaces, from which oozing was perceived, except the integumental edges, which, after stuffing the cavity left above the mouth with wads of lint, were brought together, and thus retained by four fine and two hare lip gold needles, and the twisted suture, with intervening adhesive strips.

The patient bore the operation with great firmness, having been put under the influence of anæsthetic agents in the commencement of the operation only. The amount of blood lost did not exceed twelve ounces. The action of the heart was depressed, but complete syncope did not occur. Her pulse soon rallied after the operation, when she was carried to bed. There was a little reactionary hæmorrhage, which was controlled by pressure upon the facial artery where it mounts over the lower jaw, and the application of cold water. Anodynes were administered to allay pain and procure sleep.

No untoward symptoms were manifested during the after treatment. The pulse never exceeded 90 beats in a minute, and no excited action beyond that necessary to repair was at any time observed.

25th.—The four small needles were removed to-day and some of the lint wads.

27th.—All the remaining needles and wads were now removed, and union by the first intention found to exist throughout the entire extent of the wound. Adhesive strips were re-applied, and other wads of lint, saturated with Creasote water, introduced to obviate the fœtor of the discharge.

On the 30th, precisely seven days after the operation, the patient was taken to her home in South Camden in a carriage, and the case has progressed favorably since—there being but very little deformity of the face perceptible.

*Appearance of the removed Bone and Tumor.*—The outlines of the bony mass were perfectly normal. All the cut surfaces were unchanged in structure. The fibrous degeneration or enchondroma composing the tumor extended into the antrum and filled it, with the exception of the posterior and outer fossa above the dens sapientiæ. The anterior surface, the inferior spongy bone and the nasal wall are involved and lost in the adventitious formation. The ultimate structure of the tumor having been submitted to microscopic examination by Prof. F. G. Smith, it was found to consist of fibrous filaments and cells, free from any admixture of malignant corpuscles.

*October* 27.—In the case (No. VIII.) of Mary W——, previously operated upon, (Oct. 9th,) for disease of lachrymal sac, the style was removed, then cleansed and returned, after the duct had been injected with the following:

℞. Creasoti gtt. vi.
Aquæ f.℥j.

CASE XXIV.—*Acute tonsillitis.*—Mary R——, aged 21, was affected a week since, with general fever, and pain and swelling in throat from enlarged tonsils. She was purged with a solution of Sulphate of Soda and Tartarized Antimony, and the inflammation was thus reduced to the subacute stage, in which it has been kept until this time. The same general treatment was ordered, and the application of solid Nitrate of Silver, was made to the enlarged glands.

CASE XXV.—*Strabismus Convergens* of left eye, the result of accident.—John W——, aged 24, fell with his face in a gutter, at three years of age, since which time the sight in it has been very poor. This case, then, is not congenital; and Dr. Gilbert questions whether strabismus is ever really congenital, believing that it comes on soon after birth as the result of some one of the accidents or diseases common to children. In the case in point there was, in all probability, concussion of the brain from the fall, and perhaps a slight effusion of blood, convergent strabismus supervening.

The *proximate* cause of this affection, it was further remarked, is shortening of one of the four recti muscles of the globe of the eye, but the oblique are not affected, and should never be divided. The shortening of the internal muscle is the most common. Next in order of frequency we have the external muscle shortened, producing *leer* eye. In these, as in the other cases, the opposing muscle is lengthened, the affected eye loosing its power of vision from the want of use which is induced by its unnatural position.

The cure is effected by dividing the shortened tendon, and many instruments have been employed for this purpose. Dr. Gilbert closes the sound eye by a monocle, thus giving great command over the affected organ, and relies totally on the fingers of assistants for the separation of the lids, then picking up the conjunctiva with the forceps he snips it with the curved scissors, introduces the silver hook, draws forward the tendon and divides with the angular scissors. This latter step he repeats until every offending portion of the tendon is cut and the eye occupies its proper position, since the hook, instead of passing completely beneath the tendon, may go between its parallel planes of fibres and a partial division only occurring, partial relief only follows.

Dr. Gilbert has operated a great number of times since 1842, and by these precautions has found that in 19 out of 20 cases a cure is effected.

The previous application of ice water to the eye is not resorted to by this surgeon, and in the after treatment confinement of the patient during favorable weather is deemed unnecessary. The eye is directed to be used occasionally, and cold water applied if *itching* is set up. The natural ecchymosis which follows this operation is not to be confounded with inflammation, inasmuch as no pain, heat or swelling is found accompanying it. The operation as above directed was then performed with success, the affected eye resuming its proper position.

30th.—Third day after operation; ecchymosis of the conjunctiva exists, but no inflammation; the wound has healed; the eye is perfectly straight, and moves with its fellow. The cure is established.

*October* 30th.—Case XXVI. *Encysted Tumors of Face.*—Edmund W——, aged 33. Two or three years since, small tumors began to appear upon various parts of his face, and gradually enlarged, so that now, just outside of the right external canthus of the eye, there are five of these of sufficient size to warrant removal. Such tumors will be found filled with atheromatous matter, and arise from the closure of the ducts of the sebaceous follicles of the skin, these then enlarge by the pressure of the confined secretion which soon becomes morbid in its character, the surrounding cellular tissue is made firm and resisting, forming a sac, several of which individual sacs may be agglutinated, as in this instance where three are in close contact. Dr. Gilbert has removed five or six from the face, arm and scalp of a lady who had an immense number of smaller ones over her body. These tumors are associated with a morbid condition of the skin generally, and in such cases we find *acne punctata* as a concomitant and probably a precursor.

To remove these tumors a simple incision was made on the sacs which were dissected out with their contents. The wounds were dressed in the usual manner.

CASE XXVII.—*Tenotomy* performed for *obstinate flexion of right carpus*, as a result of scarlatina, producing shortening of some of its tendons. The flexor carpi radialis,—ulnaris, and the palmaris longus seem to be at fault, producing the same deformity which we perceive in burns of the anterior part of the fore-arm and hand. In these two cases, however, the treatment is different; in the deformity from *burn* we transplant a piece of integument which takes the place of the contracted cicatrix which we cut out; but in *this case* we merely divide the *tendons* which are here at fault, and then extend the hand on a splint. The third and fourth fingers are also firmly flexed on the palm in consequence of an abscess which occurred in that place at the same time. The difficulty here resides in the palm, and will be remedied at a future period.

The narrow tenotomy knife was now introduced through the skin on the inside of the flexor carpi ulnaris and passed between this tendon and the skin; the edge of the knife was then turned upon the tendon and it was divided. The same was done on the radial side, for the division of the tendons of the palmaris longus and flexor carpi radialis. The hand was extended and cold water dressings applied.

Nov. 3d.—First dressing. The radial incision has healed by first intention, but the ulnar is granulating up; the hand is well extended and cold water dressing continued.

6th.—Ulnar incision still granulating; extension and cold appliances kept up.

10th.—Improving; full extension is borne without pain.

*November 3d.*—A case of *Congenital Teleangiectasy* in John G——, aged 20, was exhibited, and the history related, with remarks upon the disease, and the method of operation detailed. This patient will be brought before the class again for operation on the 20th inst.

Case XXVIII.—An injury by a circular saw (which was 33 inches in diameter, and whose teeth were 1 inch in length) was now presented. This accident occurred two weeks back, and as its result there was found a lacerated and contused wound of a part of the right hand. This, which involved several of the fingers, demanded an amputation of the two last phalanges of the little finger, which was dressed with one twisted suture and adhesive strips. There was also an entire separation of the palmar aspect of the ulnar side of the hand for a space of about two inches in length and one and a half in width, the presenting surface being lacerated and contused. This Prof. Gilbert made an attempt to heal by *scabbing*. To show the result of this method the patient is now brought before the class. An *artificial scab* was formed by means of Liston's isinglass plaster applied to the wound, leaving a small opening for the exit of any purulent matters at its proximal extremity; this plaster was then coated with Collodium and lint. The case is to-day dressed for the first time, and the wound is found to be almost entirely healed; the artificial scab was re-applied. The other wounds are perfectly closed.

10th.—Artificial scab removed and the entire surface beneath found cicatrized.

Case XXIX.—*A bite of mid-finger* of right hand. An Irishman, aged 21, received this injury during Election week. The case appears to have been treated for the preservation of the last phalanx, which seems only to have been originally involved, but inflammation having been set up the bone has become necrosed, the joint is invaded by the extension of the inflammation, and the patient presents himself with an enlarged, sloughing and offensive sore occupying the position of the bite.

It will be necessary here to remove the extremity of the finger in the continuity of the second phalanx away from any existing inflammation, or necrosis may again follow. Amputation was then resorted to. Two collateral digital arteries were ligated, and the flaps brought together by two sutures and adhesive strips. Cold water dressing applied.

6th.—Dressed for the first time,—doing well.

10th.—All the ligatures and sutures removed, and cerate dressing substituted for the cold water. No inflammation.

17th.—Considerable inflammation found to exist;—stump swollen, red and painful; abscess threatened; this trouble arising from the irregularity of the patient's habits. Creasote was applied as a counter-irritant over the inflamed integumental surface and cold water ordered.

20th.—Inflammation and swelling has subsided; the skin is thrown into wrinkles; re-applied the creasote.

*November* 6th.—J. S., the case (No. VII.) some time previously (Oct. 6th) operated upon for Cataract by the method for absorption, was shown, and observed to be progressing favorably. The dark ring formed by the shadow of the pupillary margin of the iris upon the lens is increasing, showing that absorption is going on with its work and its surface is receding. Prof. Gilbert here stated that after complete removal of the lens, glasses are not to be worn for some length of time, say for two or three months, and then but sparingly for a further period, since the concentration of light upon an eye previously in total darkness will act as an abnormal stimulus to the nerve, and produce all the injurious effects of such stimuli.

Case XXX.—*A Tumor of upper eyelid* of the right side, of the size of a pigeon's egg, in a negro,—Evan ——, aged 28,—was removed by making an incision over the sac, parallel to the edge of the tarsal cartilage, and then dissecting it out. The wound was closed with one gold needle, and two strips of isinglass plaster carried from the forehead to the cheek. The cold water dressing was applied.

10th.—The needle was removed on the 7th, and union by the first intention is perfect. The lid has regained almost entirely its natural size and appearance.

*November* 10th.—Case XXXI. *Chronic inflammation of external meatus of Ear.*—R. B., mulatto, aged 21. Three years since first perceived a discharge from both ears after bathing, and this has been kept up with occasional evacuations of lumps of hardened cerumen until very recently, but there has been no co-existent deafness. Upon examination with the speculum, the parietes of the external auditory meatus were found to be in a state of chronic inflammation; but the membrana tympani was healthy, which accounts for the absence of deafness. The treatment ordered was counter-irritation behind the ear by creasote, and the use of the following prescription:

℞ Cupri sulphat. gr. ij.
Aquæ fontis f.℥ j.

M. S. Inject the ear once daily after cleansing it well with warm water.

Case XXXII.—*Chronic inflammation of lachrymal sac, first stage.*—Daniel D——, aged 16. In this case there is found stillicidium lachrymarum, tumor at the internal canthus, and regurgitation of tears mingled with mucus into the eye on pressure upon the lachrymal sac. Creasote was painted over the sac and ordered to be repainted every third day, as a counter-irritant; and as a collyrium this prescription was directed:

℞ Zinci sulphat. gr. j.
Aq. rosæ f℥j.

M. S. To be dropped in the eye once daily.

13th.—Improving; the tears pass more freely into the nose. Creasote re-applied and collyrium continued.

Case XXXIII.—*Ozæna* in a female, aged about 30, resulting upon secondary syphilis, which was communicated to the patient by nursing a child thus affected, her nipple being first the seat of the disease, and her constitution suffering in consequence. She was ordered

℞. Syr. sarsap. comp. Oj.
Auri chloridi gr. j.

M. S. A tablespoonful thrice daily.

*November* 13th.—Case XXXIV. *Loss of right eye from wound.* John K——, aged 22, a machinist, whilst engaged in his occupation, was struck in the eye by a chip of iron, which wounded the globe, but was then supposed to have rebounded, inasmuch as the wound healed readily with the ordinary result of such injuries; in three weeks, however, a black speck presented at the wounded point, which speck was found to be a foreign body, and this being discharged proved an iron turning measuring a half by five-eighths of an inch in its superficial diameter aud several lines in thickness, weighing 72 *grains*. Probably the largest foreign body ever lodged in the eye, without producing anything beyond ordinary symptoms; the cushion-like resistance of the internal structures of the globe no doubt having prevented its passage into the cranium.

An artificial eye can be readily fitted upon the resulting stump, which is good.

CASE XXXV.—*Bite of Thumb.*—Mary M——, aged 14. This, the second case of wound of this character brought before the class within ten days, has resulted in the milder form of paronychia only, namely: abscess at the root of the nail, which is separating by the use of poultices.

CASE XXXVI. *Operation for occlusion of lachrymal duct on left side.*—Mrs. C., aged about 50, has had chronic inflammation of lachrymal sac and duct for nine years, with all the usual concomitant symptoms, and now presents herself for relief, the duct being totally occluded. The ordinary operation was performed, and a bougie made before the class by rubbing white wax upon a strip of fine linen, and then rolling it up conically, was introduced.

17th.—The bougie was removed and the silver style introduced.

*Nov.* 17th.—This morning Prof. Gilbert presented a tumor of the right breast which he had removed on Monday the 15th, from a female patient, aged 38. It was first noticed about four years ago, and was then supposed to have arisen from a contusion, since which time it had gradually increased. Its external appearance was irregularly lobulated, and its feel very much like that of a number of small oranges in a bag. There was no discoloration of the integument, which was perfectly moveable, and the nipple was not retracted. The disease appeared under 40 years of age, before which period tumors of this kind are almost never malignant. It was of slow growth and painless. All these facts led to the belief that it was a Cystic sarcoma, which is benign, and thus its removal was undertaken. Amputation was performed, and the entire mass, whichwas found to weigh 7 lbs. 6 oz., turned out of its bed upon the pectoralis major muscle.

Very little of the mammary gland is observed as remaining; the cellular and fibrous envelopes of its lobules being greatly developed, and forming with several large sinuous serous cysts the mass of the tumor. Some of the fluid discharged from the cysts was microscopically examined by Prof. F. G. Smith of this Institution, and the diagnosis of the benignity of the tumor confirmed. The result of his investigations are as follows:

1st. Pale, very transparent cells. 1-50th to 1-100th of a line in diameter, the majority containing pale nuclei.

2d. Tabular plates of cholesterine.

3d. Small round corpuscles about 1-250th of a line in diameter, resembling pus corpuscles, and cells of epithelium newly formed.

4th. Globules resembling milk.

The fluid is alkaline in its re-action, glutinous in consistence, and contains a large quantity of albumen, as evidenced by coagulation with heat.

There are no cells of malignant character discoverable.

20th.—The patient is reported as doing remarkably well.

Case XXXVII.—*The results of strumous inflammation of Knee joint,* in Thomas R——, aged 17, were stated to have been false anchylosis of the joint, with the leg flexed almost at a right angle upon the thigh from contraction of the tendons of the inner and outer hamstring muscles, together with firm adhesions of the integument to the bones and fasciæ about the joint. Subcutaneous section of the inner and outer hamstrings was made by the late Prof. Grant in conjunction with Prof. Gilbert about 18 months since, and the limb extended forcibly. The patient has had good use of his limb for now nearly six months, and is brought forward to prove the value of Tenotomy as a remedial agent in the treatment of deformities.

## Figures and Tables

**Figure f1:**